# Clinical characteristics, microbiological landscape, and severity-stratified management of pyogenic spondylitis: a retrospective cohort study

**DOI:** 10.3389/fcimb.2026.1883513

**Published:** 2026-08-28

**Authors:** Zongqiang Yang, Qiang Liu, Junxiao Liu, Hanhua Guo, Zhangui Gu, Long Ma, Ningkui Niu, Jiandang Shi

**Affiliations:** 1 Department of Orthopedics, General Hospital of Ningxia Medical University, Yinchuan, Ningxia Hui Autonomous Region, China; 2 The First Clinical Medical College of Ningxia Medical University, Yinchuan, Ningxia Hui Autonomous Region, China

**Keywords:** debridement, microbiological yield, posterior fixation, pyogenic spondylitis, severity stratification, spinal infection, tiered management

## Abstract

**Objective:**

To characterize the clinical, radiological, and microbiological features of pyogenic spondylitis (PS) and to describe outcomes observed within a severity-stratified real-world management pathway integrating antimicrobial therapy, stabilization, debridement, and fusion according to structural instability and neurological risk.

**Methods:**

We conducted a retrospective observational cohort study of 149 patients with PS treated at Ningxia Medical University General Hospital between January 2015 and December 2023. Patients were retrospectively grouped according to baseline structural severity, neurological status, and the management strategy actually used in routine care: Group A, antimicrobial therapy plus external immobilization (n = 48); Group B, posterior fixation and fusion plus antimicrobial therapy (n = 57); and Group C, posterior fixation with debridement and fusion via posterior and/or anterior approaches plus antimicrobial therapy (n = 44). Clinical characteristics, microbiological findings, inflammatory markers, VAS, ODI, Cobb angle, perioperative variables, complications, and endpoint-defined recovery were analyzed. Principal-component analysis and adjusted response models were prespecified as exploratory analyses.

**Results:**

The three groups represented distinct severity strata, ranging from structurally stable disease to destructive disease with neurological compromise. Final diagnostic adjudication identified 73 microbiologically confirmed cases, whereas the prespecified patient-level yield analysis was limited to a 66-case interpretable pretreatment subset, within which positivity was lower in Group C than in Groups A and B (40.0% vs 82.4% and 84.2%, respectively; P = 0.001). At the specimen level, 95 bacterial isolate records were summarized, and *S. aureus* was the predominant isolate (48/95). All groups showed improvement in inflammatory, pain, functional, and radiological measures over follow-up, although the tempo of recovery differed across severity-matched management strata. Exploratory adjusted analyses were directionally consistent with these descriptive patterns but should be interpreted cautiously.

**Conclusions:**

In this retrospective real-world cohort, a severity-stratified management pathway for PS was feasible and showed coherent microbiological, inflammatory, functional, and radiological recovery patterns within each clinical stratum. These findings support pragmatic escalation according to structural instability and neurological risk, but they should not be interpreted as comparative evidence of treatment superiority.

## 1 Introduction

Pyogenic spondylitis (PS) is an infectious inflammatory disease involving the vertebral bodies, intervertebral discs, adjacent endplates, and paravertebral soft tissues caused by pyogenic bacterial pathogens. It represents a major form of adult spinal infection and continues to impose substantial morbidity in contemporary practice (Conti et al., 2025; Placide and Reznicek, 2025). Recent epidemiological studies indicate that the incidence of infectious spondylodiscitis is increasing, particularly among older adults and patients with complex medical comorbidities or prior invasive procedures (Son et al., 2023; Thavarajasingam et al., 2023a; Conti et al., 2025; Henry et al., 2025; Motoyoshi et al., 2025).

Although PS is classified as a bacterial spinal infection, its clinical course extends beyond infection alone and often includes vertebral destruction, segmental instability, deformity, and neurological risk. Recent cohorts consistently show that the lumbar spine is most commonly affected and that middle-aged to elderly men with diabetes, malnutrition, renal disease, or other immunocompromising conditions constitute the predominant patient population (Pantel et al., 2024; Conti et al., 2025; Henry et al., 2025). However, early clinical presentation remains nonspecific. Localized back pain is common, whereas fever and marked leukocytosis are inconsistently observed, contributing to diagnostic delay and more advanced disease at presentation (Son et al., 2023; Conti et al., 2025; Sato et al., 2025).

Imaging and microbiological evaluation constitute the cornerstone of differential diagnosis in PS. Magnetic resonance imaging typically shows involvement of adjacent vertebral endplates and the intervertebral disc, with associated paravertebral or epidural inflammatory extension or abscess formation (Maamari et al., 2022; Crombe et al., 2024). At the same time, other infectious and non-infectious conditions, including tuberculous spondylodiscitis and degenerative or inflammatory mimics, must be considered during image interpretation (Piccolo et al., 2024). From a microbiological perspective, *Staphylococcus aureus* remains the most frequently isolated pathogen, followed by streptococci and Gram-negative bacilli (Froschen et al., 2024; Hijazi et al., 2024). Blood cultures and image-guided or surgically obtained tissue samples remain central to pathogen-directed treatment, although microbiological yield varies with clinical context and sampling strategy (Maamari et al., 2022; Hijazi et al., 2024; Haykir Solay et al., 2025).

Given its insidious presentation, diagnostic complexity, and destructive nature, the optimal management of PS remains controversial. In recent years, international literature has increasingly emphasized severity-based and stability-oriented treatment frameworks for spinal infection (Campbell et al., 2023; Pluemer et al., 2023; Bigdon et al., 2025; Rawall et al., 2025). These approaches stratify patients according to structural instability, abscess burden, neurological status, and systemic condition, while also recognizing failure of medical treatment as an important trigger for escalation of care (Urrutia et al., 2024). In carefully selected mild cases, antimicrobial therapy with immobilization may be sufficient, whereas fixation, debridement, decompression, and reconstruction become increasingly relevant as structural destruction and neurological compromise progress (Campbell et al., 2023; Thavarajasingam et al., 2023b; Bigdon et al., 2025; Rawall et al., 2025). Nevertheless, much of the current evidence remains retrospective and heterogeneous, and the optimal intervention strategy for patients with intermediate disease severity is still incompletely defined.

Against this background, the present study aimed to systematically analyze the clinical characteristics, imaging features, microbiological landscape, and severity spectrum of PS, and to internally evaluate a clinically applicable stratified intervention strategy based on pain severity, radiological destruction, mechanical stability, and neurological risk. Using real-world clinical data, we sought to clarify whether severity-matched treatment was associated with coherent inflammatory, functional, radiological, and safety outcomes.

## 2 Materials and methods

### 2.1 Clinical data

**Figure 1:**
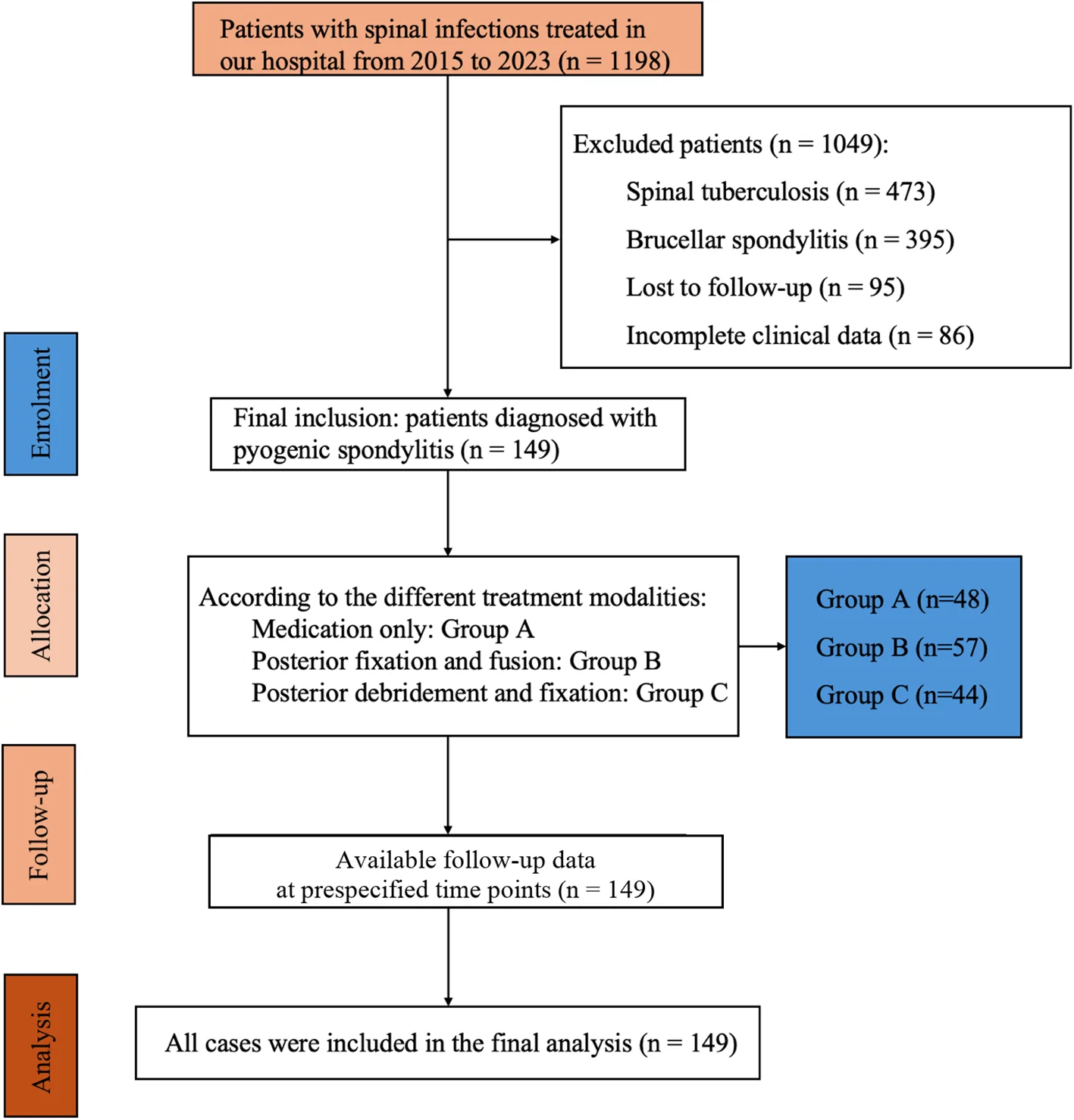
Patient screening and retrospective severity-stratified management allocation. Clinical records of 1,198 patients treated for spinal infection between January 2015 and December 2023 were screened. Exclusions were spinal tuberculosis (n = 473), brucellar spondylitis (n = 395), lost to follow-up (n = 95), and incomplete clinical data (n = 86). The final cohort comprised 149 patients with pyogenic spondylitis, who were allocated retrospectively into Group A, Group B, or Group C according to structural severity, instability, abscess burden, and neurological risk.

This retrospective study screened the clinical records of 1,198 patients with spinal infections who were admitted to Ningxia Medical University General Hospital between January 2015 and December 2023. As shown in **Figure 1**, cases ultimately identified as spinal tuberculosis or brucellar spondylitis were removed during the screening stage before pyogenic cohort inclusion. After final diagnostic adjudication and analytic eligibility review, 149 patients with pyogenic spondylitis (PS) were ultimately enrolled for analysis.

Inclusion criteria were as follows:

1. Age >=18 years;
2. Admission to our institution during the study period from January 2015 to December 2023;
3. A final diagnosis of PS established by microbiological confirmation or by integrated pathological and clinicoradiological assessment after exclusion of infectious and non-infectious mimicking conditions, consistent with contemporary diagnostic frameworks (Maamari et al., 2022; Crombe et al., 2024; Piccolo et al., 2024; Rawall et al., 2025).

Post-adjudication analytic exclusions included:

1. Incomplete clinical or imaging records that precluded severity stratification or outcome assessment;
2. Loss to follow-up before the prespecified outcome assessment time points.

This retrospective study was approved by the Ethics Committee of Ningxia Medical University General Hospital (approval No. KYLL-2026-0052; approval date: January 28, 2026). Because this study analyzed previously completed routine clinical care from January 2015 to December 2023, it was reviewed as a retrospective chart-based cohort rather than as a prospective interventional trial. The requirement for individual written informed consent was waived by the Ethics Committee for this retrospective review, and all procedures were conducted in accordance with the Declaration of Helsinki. Because no prospective intervention allocation was performed, clinical trial registration was not applicable to this retrospective observational cohort.

### 2.2 Study design

Clinical trial registration: not applicable, because this study was a retrospective observational cohort without prospective assignment of interventions.

#### 2.2.1 Diagnostic criteria

The diagnosis of PS was established based on a combination of clinical, microbiological, and pathological evidence.

Clinical diagnostic criteria included localized spinal pain with or without systemic symptoms such as fever; CT and/or MRI findings demonstrating vertebral body, intervertebral disc, or paravertebral soft-tissue involvement with bone destruction or abnormal signal intensity, with or without abscess formation; and exclusion of common mimicking conditions.

Microbiological confirmation was sought before antimicrobial escalation whenever clinically feasible by blood cultures and/or pathogen testing of pus, bone tissue, or paravertebral soft-tissue specimens obtained through percutaneous biopsy or surgery. Conventional bacterial cultures were performed routinely, and metagenomic next-generation sequencing (mNGS) was used selectively when available or when conventional testing remained nondiagnostic. In urgent cases requiring immediate antimicrobial treatment or surgery, care was not delayed solely to complete every microbiological test before intervention.

Pathological evidence consisted of histological features consistent with pyogenic inflammation, such as neutrophilic infiltration and tissue destruction.

A diagnosis of PS was established by microbiological confirmation when available, or by compatible clinicoradiological features, exclusion of mimicking diseases, and supportive pathological findings when microbiological tests were negative, nonspecific, or unavailable, consistent with current diagnostic frameworks (Maamari et al., 2022; Rawall et al., 2025). Response to antimicrobial therapy was treated as supportive follow-up information rather than a primary diagnostic criterion.

#### 2.2.2 Grouping criteria

Patients with PS were managed retrospectively within a severity-stratified clinical pathway based on disease burden, spinal structural stability, and neurological status. The three groups therefore represent real-world management strata rather than prospectively assigned trial arms. The operational thresholds used below were pragmatic institutional criteria informed by contemporary spinal-infection frameworks and score-based literature (Campbell et al., 2023; Pluemer et al., 2023; Bigdon et al., 2025; Britt et al., 2025; Rawall et al., 2025) rather than a prospectively validated scoring system:

Medical treatment group (Group A, n = 48): Patients received external brace immobilization combined with systemic antimicrobial therapy. Imaging demonstrated only mild involvement of the vertebral endplates or intervertebral discs, with no vertebral collapse or kyphotic deformity (Cobb angle <10°). Abscesses were absent or minimal (paravertebral abscess <1–2 cm without epidural compression). No neurological deficits were present [American Spinal Injury Association (ASIA) Impairment Scale grade E], and patients were systemically stable without evidence of sepsis.

Posterior fixation and fusion group (Group B, n = 57): Patients underwent posterior pedicle screw fixation with posterolateral bone graft fusion in addition to systemic antimicrobial therapy. Vertebral destruction was evident but did not result in severe collapse. Moderate kyphotic deformity was present (Cobb angle 10°–30°). Moderate-sized paravertebral or intraspinal abscesses were observed, with possible epidural involvement but without significant neurological deficits. Patients experienced pronounced pain, particularly mechanical pain, and mild neurological symptoms such as intermittent radicular pain or sensory disturbances. Clinical symptoms were persistent or unstable despite antimicrobial treatment.

Posterior fixation with debridement and fusion group (Group C, n = 44): Patients underwent posterior pedicle screw fixation combined with thorough debridement and bone graft fusion, along with systemic antimicrobial therapy. These patients exhibited severe vertebral collapse and marked kyphotic deformity (Cobb angle ≥30°). Large paravertebral or epidural abscesses were present, occupying more than 50% of the spinal canal or causing spinal cord and/or nerve root compression. Significant neurological deficits were observed [ASIA grades A-D], and systemic infection was severe, including bacteremia, systemic inflammatory response syndrome (SIRS), or sepsis, often with multilevel spinal involvement.

#### 2.2.3 Treatment protocols

At presentation, blood cultures and other accessible diagnostic specimens were obtained whenever clinically feasible before empirical antimicrobial treatment. When blood cultures were nondiagnostic and the patient remained sufficiently stable, CT-guided or percutaneous biopsy was pursued before definitive escalation whenever feasible. mNGS was used selectively when conventional testing remained nondiagnostic or when additional pathogen clarification was clinically necessary. Empirical intravenous therapy was then initiated using vancomycin combined with a third-generation cephalosporin or a fluoroquinolone. In patients managed surgically, the interval from admission evaluation to surgery depended on structural instability, neurological compromise, and response to early medical stabilization; intraoperative tissue specimens were again collected for histopathological examination, conventional culture, and mNGS when available. After pathogen identification, antimicrobial regimens were adjusted according to susceptibility testing. Within the institutional pathway summarized in **Supplementary Table 2**, the planned antimicrobial durations were 12 weeks in Group A (6 weeks intravenous plus 6 weeks directed therapy), 10 weeks in Group B (4 weeks intravenous plus 6 weeks directed therapy), and 12 weeks in Group C (6 weeks intravenous plus 6 weeks directed therapy), with pathogen-specific adjustment when clinically required. Exact day-level intervals between admission, sampling, mNGS reporting, and surgery were not analyzed as formal quantitative endpoints in this retrospective cohort.

##### 2.2.3.1 Medical treatment group

Patients in the medical treatment group received empirical intravenous antimicrobial therapy (vancomycin combined with a third-generation cephalosporin or a fluoroquinolone) for 6 weeks, followed by 6 weeks of pathogen-directed or empirically adjusted antimicrobial therapy. The planned total treatment duration in this pathway was 12 weeks. External brace immobilization and bed rest were implemented during treatment. Serum inflammatory markers (CRP and ESR) and magnetic resonance imaging (MRI) were periodically reassessed to monitor disease progression and treatment response.

##### 2.2.3.2 Surgical treatment group

Surgical treatment included a posterior fixation and fusion group (Group B) and a posterior fixation combined with lesion debridement and fusion group (Group C). All surgical procedures were performed under the supervision of the same senior spine surgeon to minimize inter-operator variability.

###### 2.2.3.2.1 Posterior pedicle screw fixation with posterolateral fusion

For Group B, posterior fixation and fusion were performed through a standard posterior approach. Pedicle-screw instrumentation was placed under fluoroscopic guidance, deformity correction was applied as needed, and posterolateral/interlaminar fusion was completed using allograft or synthetic bone. Intraoperative specimens obtained by aspiration or curettage were sent for histopathological examination, conventional microbiological culture, and mNGS when available. Formal anterior-column debridement was not routinely performed in this group because these patients primarily required stabilization for focal instability rather than the degree of destructive burden or major neural compression that mandated radical debridement-based reconstruction.

###### 2.2.3.2.2 Posterior pedicle screw fixation with posterior debridement and fusion

For Group C patients who could be adequately managed through a posterior-only approach, posterior decompression, debridement of infected and necrotic tissues, interbody structural support, and fixation were performed under fluoroscopic guidance. Debrided tissues were submitted for histopathological examination, conventional microbiological culture, and mNGS when available.

###### 2.2.3.2.3 Posterior pedicle screw fixation with anterior debridement and fusion

When anterior-column destruction, paravertebral or epidural burden, or reconstruction demands exceeded what could be addressed posteriorly, a combined posterior-anterior strategy was used. Posterior pedicle-screw fixation was first applied for preliminary stabilization, followed by an anterior approach for radical debridement and interbody reconstruction with tricortical iliac graft or titanium mesh filled with autologous bone as appropriate. All debrided tissues were sent for histopathological examination, bacterial culture, and mNGS when available.

##### 2.2.3.3 Postoperative management

Standard postoperative spinal care was provided, with particular emphasis on nutritional support. Surgical drains were removed when the drainage volume was less than 50 mL per day. Patients remained on bed rest for 2 weeks and subsequently ambulated with external brace support. Postoperative antimicrobial therapy was adjusted according to confirmed microbiological results. In the institutional treatment pathway summarized in **Supplementary Table 2**, Group B generally received 4 weeks of intravenous therapy followed by 6 weeks of directed therapy, whereas Group C generally received 6 weeks of intravenous therapy followed by 6 weeks of directed therapy.

#### 2.2.4 Outcome measures

The following variables were collected and analyzed:

1. Baseline clinical characteristics: age, sex, disease duration, comorbidities (diabetes mellitus, hypertension, history of corticosteroid use), symptom duration, smoking and alcohol consumption history, affected spinal region and segments, and abscess distribution.
2. Laboratory parameters included complete blood count, C-reactive protein (CRP), erythrocyte sedimentation rate (ESR), procalcitonin (PCT), and serum albumin (Alb). These markers were selected to reflect systemic inflammatory burden, response to infection control, and general nutritional/inflammatory status during recovery.
3. Pain and functional outcomes: Oswestry Disability Index (ODI) and Visual Analog Scale (VAS).
4. Neurological status: Neurological deficits at admission were assessed using the American Spinal Injury Association (ASIA) Impairment Scale. ASIA grades A-D were defined as neurological impairment, whereas ASIA grade E was defined as no neurological deficit.
5. Perioperative outcomes: operative time, intraoperative blood loss, transfusion rate, length of hospital stay, time to ambulation (days), and intensive care unit (ICU) admission.
6. Radiological evaluation: Changes in Cobb angle were measured on plain radiographs before and after treatment. Computed tomography (CT) was used to assess the location and number of affected vertebrae, presence of skip lesions, and degree of vertebral collapse. The estimated original height of the affected vertebra or intervertebral space was calculated as the mean height of the two adjacent unaffected vertebrae or disc spaces. Collapse greater than one-third of the estimated original height was classified as severe, whereas collapse of one-third or less was classified as mild. MRI was used to evaluate epidural and paravertebral abscesses. In the surgical groups (Groups B and C), bony fusion and instrumentation status (loosening or breakage) were assessed using the Bridwell fusion grading system (grades I-IV). In the medical treatment group (Group A), radiological stability was evaluated based on maintenance of vertebral height and intervertebral space. Clinical and laboratory outcomes were summarized at baseline and at approximately 1, 3, and 6 months when data were available, whereas radiographic follow-up in the surgical groups was summarized separately because postoperative imaging intervals were not uniform.
7. Complications included surgical site infection, cerebrospinal fluid leakage, antibiotic-related liver injury, recurrent abscess or recurrent infection, reoperation, and infection-related death. Recurrence was defined as renewed clinical or imaging evidence of active infection after initial improvement, and treatment failure was defined as persistence or progression requiring escalation of management.

#### 2.2.5 Exploratory microbiological, inflammatory, and response analyses

For final diagnostic adjudication, cases were classified as microbiologically confirmed when an etiologic pathogen was identified by blood culture, conventional culture of biopsy/surgical specimens, or clinically adjudicated metagenomic next-generation sequencing (mNGS) at any point in the diagnostic workup. Separately, the prespecified patient-level yield analysis was intentionally restricted to a single pretreatment microbiological testing episode that could be classified unambiguously as positive or negative, because this definition was designed to compare early diagnostic yield across management strata without inflating the surgically treated groups through repeated or intraoperative sampling obtained later in the workup. “Positive” indicated recovery or detection of an etiologic pathogen in that pretreatment episode, “negative” indicated completed pretreatment testing without pathogen detection, “nonspecific” indicated contaminant flora or indeterminate findings that could not support binary adjudication, and “missing/unavailable” indicated that no classifiable pretreatment microbiological result was available in the chart. Only positive and negative pretreatment records were entered into the binary yield denominator. To avoid mixing denominators, specimen-level bacterial isolate records were summarized separately and were not used as the denominator for patient-level positivity.

To explore whether systemic inflammatory activity paralleled structural disease severity, baseline WBC, neutrophil percentage, CRP, ESR, PCT, albumin, and IL-6 were integrated using principal component analysis. Missing laboratory values were imputed using variable medians, albumin was direction-reversed so that higher integrated scores reflected greater inflammatory burden, and the first principal component was retained as an exploratory inflammatory-burden score for comparisons across severity strata.

Clinically interpretable dynamic response endpoints were additionally calculated to enrich treatment-response assessment. These included early CRP response (>=50% reduction from baseline at 1 month), ESR normalization (<20 mm/h at 3 and 6 months), clinically meaningful pain response (VAS reduction >=2 points at 1 month), favorable final function (ODI <=20%), and final radiological stability or correction (final Cobb angle no greater than baseline). For exploratory continuous visualization, laboratory percentage change was calculated as 100 × (baseline - follow-up)/baseline; positive values therefore indicate reduction and negative values indicate interval increase. No winsorizing or capping was applied, so patients with very low baseline values or marked early rebound could yield values below -100%. These threshold-based summaries were used descriptively to complement the serial continuous outcome analyses.

To determine whether continuous treatment-response differences persisted after adjustment for baseline covariates, exploratory standardized linear models were fitted for CRP percentage change at 1 month, ESR percentage change at 3 months, VAS reduction at 1 month, final ODI improvement, and final Cobb correction. Models adjusted for age, sex, diabetes, paravertebral abscess, pretreatment Cobb angle, and baseline values when applicable. Bootstrap 95% confidence intervals were used to summarize the precision of adjusted contrasts. Because the continuous laboratory percentage-change variables were exploratory and sensitive to baseline-ratio effects, these adjusted contrasts were interpreted cautiously and were not treated as primary inferential endpoints.

### 2.3 Statistical analysis

All primary statistical analyses were performed using SPSS software (version 25.0; IBM Corp., Armonk, NY, USA), and exploratory multivariable and figure-generating analyses were performed in R (version 4.4.2). Continuous variables were tested for normality. Normally distributed data were expressed as mean +/- standard deviation and compared using one-way analysis of variance, followed by Tukey’s *post hoc* test when appropriate. Non-normally distributed data or data with unequal variances were expressed as median (25th-75th percentiles) and compared using the Kruskal-Wallis H test, followed by Dunn’s test with multiple-comparison correction. Categorical variables were expressed as counts (percentages) and compared using the chi-square test or Fisher’s exact test, as appropriate. Because follow-up completeness varied across endpoints and this was a retrospective cohort, time-point comparisons were interpreted descriptively. PCA-derived inflammatory-burden scores, binary response endpoints, and adjusted continuous response contrasts were prespecified as exploratory analyses. All tests were two-sided, and P < 0.05 was considered statistically significant.

## 3 Results

### 3.1 General clinical characteristics

A total of 149 patients with pyogenic spondylitis (PS) were included in this study, comprising 48 patients in Group A, 57 in Group B, and 44 in Group C. The overall cohort was predominantly composed of middle-aged and elderly males (male/female ratio: 101/48) and demonstrated a substantial burden of comorbidities. Diabetes mellitus was present in 46 patients (30.9%), hypertension in 51 (34.2%), a history of smoking in 29.5%, and alcohol consumption in 26.2%. In addition, 24 patients (16.1%) had a history of prior spinal surgery, 15 (10.1%) had a history of spinal puncture, 12 (8.1%) were receiving hemodialysis, and 18 (12.1%) had a history of corticosteroid use. Regarding final diagnostic adjudication, 73 patients (49.0%) achieved microbiological confirmation at some point in the workup, 31 (20.8%) had pathological support despite negative or nondiagnostic microbiology, and 45 (30.2%) were diagnosed based on integrated clinical, laboratory, and imaging findings after exclusion of mimicking disorders.

Regarding the distribution of affected spinal segments, the lumbar spine was most commonly involved (114 cases, 76.5%), followed by the thoracic spine (25 cases, 16.8%) and cervical spine (10 cases, 6.7%). Multilevel involvement was observed in 21 patients (14.1%). Imaging at admission revealed paravertebral abscesses in 35.6% of patients, endplate destruction in 97/149 (65.1%), and posterior column involvement in 11.4%. Neurological deficits of varying severity were present in 59 patients (39.6%). With respect to vertebral collapse, 32.2% of patients exhibited no collapse, 36.2% had moderate collapse, and 31.5% had severe collapse.

There were no statistically significant differences among the three groups in age, sex, or major comorbidities (*P* > 0.05). In contrast, key clinical and radiological indicators reflecting disease burden, including endplate destruction, vertebral collapse, Cobb angle, paravertebral abscess formation, and neurological deficits, showed a clear gradient across the three groups (**Table 1**). These findings indicate that the cohort captured distinct severity strata within the retrospective clinical pathway.

**Table 1:** Baseline clinical and radiological characteristics of patients in three groups.

Variable	Total	Group A (n = 48)	Group B (n = 57)	Group C (n = 44)	*P* value
Demographics					
Age (years)	59.0(51.0,69.0)	60.5(49.5,71.0)	59.0(50.0,68.0)	57.5(52.0,69.3)	0.833
Sex (M/F)	101/48	36/12	37/20	28/16	0.426
Disease duration (month)	1.0(1.0,2.0)	1.0(0.7,1.0)	2.0(1.0,2.0)**^#^**	1.0(1.0,3.0)^*^	<0.001
Diagnostic evidence					
Final microbiological confirmation at any point in routine diagnostic workup, n (%)	73 (49.0%)	—	—	—	—
Pathologically supported despite negative or nondiagnostic microbiology, n (%)	31 (20.8%)	—	—	—	—
Diagnosed by integrated clinical, laboratory, and imaging findings after exclusion of mimicking disorders, n (%)	45 (30.2%)	—	—	—	—
Comorbidities					
Diabetes (%)	46(30.9%)	13 (27.1%)	15(26.3%)	18(40.9%)	0.228
Hypertension (%)	51(34.2%)	17(35.4%)	14(24.6%)	20(45.5%)	0.088
Smoking history(%)	44(29.5%)	12(25.0%)	17(29.8%)	15(34.1%)	0.633
Drinking history(%)	39(26.2%)	10(20.8%)	16(28.1%)	13(29.5%)	0.585
History of spinal surgery(%)	24(16.1%)	4(8.3%)	9(15.8%)	11(25.0%)	0.094
Previous spinal puncture	15(10.1%)	5(10.4%)	4(7.0%)	6(13.6%)	0.643
Dialysis history(%)	12(8.1%)	2(4.2%)	5(8.8%)	5(11.4%)	0.447
Systemic corticosteroid exposure (%)	18(12.1%)	4(8.3%)	7(12.3%)	7(15.9%)	0.537
Clinical presentation					
Fever (%)	62(41.6%)	10(20.8%)	20(35.1%)^#*^	32(72.7%)^*^	<0.001
Neurologic deficit (%)	59(39.6%)	0(0.0%)	20(35.1%)^#*^	39(88.7%)^*^	<0.001
VAS score at admission	8.2 (7.8, 8.6)	8.1 (7.8, 8.5)	8.2 (7.8, 8.7)	8.4 (8.1, 8.7)	0.207
Systemic toxicity	35(23.5%)	1(2.1%)	8(14.0%)^#*^	26(59.1%)^*^	<0.001
Radiological features					
Involved region					
Lumbar vertebrae	114(76.5%)	39(81.2%)	42(73.7%)	33(75.0%)	0.634
Thoracic vertebrae	25(16.8%)	7(14.6%)	11(19.3%)	7(15.9%)	0.79
Cervical vertebrae	10(6.7%)	2(4.2%)	4(7.0%)	4(9.1%)	0.589
Degree of vertebral collapse					
No collapse	48(32.2%)	48/48	0	0	<0.001
Moderate collapse	54(36.2%)	0	50(87.7%)^#*^	4(9.1%)	<0.001
Severe collapse	47(31.5%)	0	7(12.3%)^#*^	40(90.9%)	<0.001
Cobb angle (°)	18.0(8.0,31.0)	7.0 (6.0, 8.0)	19.0(17.0,21.0)^#*^	34.5(32.0,37.0)	<0.001
Multilevel involvement	21(14.1%)	0(0%)	8(14.0%)^#*^	13(29.5%)^*^	<0.001
Paravertebral abscess	53(35.6%)	9(18.8%)	23(40.4%)^#*^	21(47.7%)^*^	<0.05
Endplate destruction	97/149(65.1%)	21(43.8%)	39(68.4%)^#*^	37(84.1%)^*^	<0.001
Posterior column involved	17(11.4%)	1(2.1%)	5(8.8%)^#*^	11(25.0%)^*^	<0.05

*P < 0.05 compared with Group A.

#*P* < 0.05 compared with Group C.

### 3.2 Laboratory and microbiological findings

At admission, inflammatory markers were markedly elevated in all three groups, while their distributions demonstrated a severity-dependent gradient consistent with disease stratification (**Table 2**). Median C-reactive protein (CRP) levels were 55.9 (53.1–75.0) mg/L in Group A, 72.6 (65.0–81.2) mg/L in Group B, and 88.6 (82.1–100.2) mg/L in Group C (*P* < 0.001). Similarly, erythrocyte sedimentation rate (ESR) values increased progressively from Group A (59.1 ± 15.4 mm/h) to Group C (73.3 ± 17.8 mm/h) (*P* < 0.001). These findings indicate that the intensity of the inflammatory response paralleled disease severity rather than treatment selection.

**Table 2:** Laboratory and microbiological findings at admission.

Parameter	Group A	Group B	Group C	*P* value
WBC (×10^9^/L)	9.8(6.7,13.0)	10.1(7.1,12.4)	8.7(7.2,11.3)	0.38
Neutrophils (%)	73.4(64.0,85.7)	78.3(66.4,86.0)	70.7(61.37,83.6)	0.21
CRP (mg/L)	55.9(53.1,75.0)	72.6(65.0,81.2)	88.6(82.1,100.2)	<0.001
ESR (mm/h)	59.10 ± 15.38	64.75 ± 18.17**^#^**	73.25 ± 17.84^*^	<0.001
PCT (ng/mL)	0.04(0.04,0.04)	0.07(0.04,0.37)^*^	0.08(0.04,0.27)^*^	<0.001
Albumin (g/L)	37.4(30.5,41.3)	32.2(28.8,35.4)^#*^	30.4(23.0,34.9)^*^	<0.001
T-SPOT	0 (0%)	0 (0%)	0 (0%)	1
Brucella test	0 (0%)	0 (0%)	0 (0%)	1
Single-episode interpretable pretreatment yield subset, positive/total (%)	14/17 (82.4%)	16/19 (84.2%)	12/30 (40.0%)	0.001
Specimen-level bacterial isolate records (n = 95)				
*S. aureus*	12	22	14	
Escherichia coli	3	5	3	
Klebsiella pneumoniae	2	2	4	
Viridans streptococci	4	3	0	
Other bacterial isolates	4	8	9	
Histology				
Neutrophilic infiltration	48(100.0%)	57(100.0%)	44(100.0%)	1
Granuloma/Caseation	0 (0%)	0 (0%)	0 (0%)	1

*P < 0.05 compared with Group A.

#*P* < 0.05 compared with Group C.

Procalcitonin (PCT) and serum albumin levels further reinforced this trend. Group A exhibited the lowest PCT levels [0.04 (0.04–0.04) ng/mL], whereas significantly higher values were observed in Groups B and C (*P* < 0.001), suggesting a greater infectious burden and extent of tissue destruction in more severe disease. In contrast, serum albumin levels declined progressively with increasing severity, from 37.4 g/L in Group A to 30.4 g/L in97/149 (65.1%) Group C (*P* < 0.001), reflecting more pronounced systemic inflammatory consumption and nutritional impairment in advanced cases.

**Figure 2:**
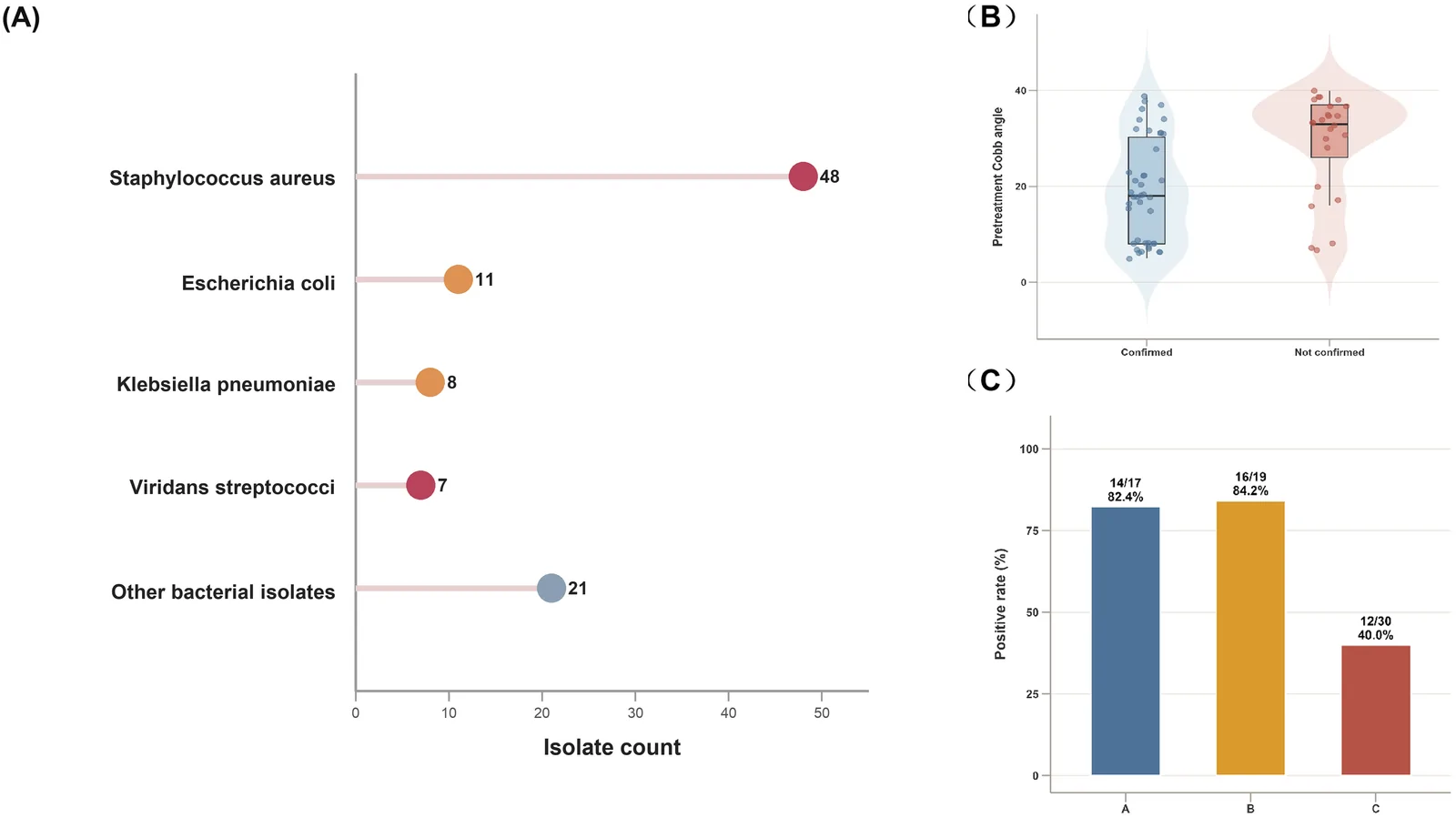
Microbiology and structural severity landscape in pyogenic spondylitis. **(A)** Specimen-level bacterial isolate spectrum. **(B)** Pretreatment Cobb angle distribution according to microbiological confirmation status. **(C)** Patient-level microbiological detection yield across treatment groups. Boxes indicate interquartile ranges, center lines indicate medians, and points indicate individual patients where shown.

Microbiological analysis provided two complementary perspectives. Final diagnostic classification and early patient-level yield were intentionally analyzed separately. Among the 149 included patients, 73 were ultimately microbiologically confirmed on the basis of blood culture, conventional culture of biopsy or surgical specimens, and/or clinically adjudicated mNGS results obtained during the diagnostic workup. However, the prespecified patient-level yield analysis was intentionally restricted to a single pretreatment microbiological episode that could be classified unambiguously as positive or negative, because including repeated or intraoperative sampling would have disproportionately favored the surgically managed groups and would not have reflected a comparable early diagnostic opportunity across strata. Within that interpretable 66-case subset, 42 records were positive and 24 were negative, yielding an overall positivity rate of 63.6%; group-specific positivity rates were 14/17 (82.4%) in Group A, 16/19 (84.2%) in Group B, and 12/30 (40.0%) in Group C (P = 0.001). The remaining 83 patients were outside this binary denominator because the available pretreatment record was nondiagnostic or unavailable; among them, 31 were subsequently microbiologically confirmed by repeated or intraoperative testing and therefore contributed to the final 73-case microbiological-confirmation count, whereas the re97/149 (65.1%)maining 52 were diagnosed through pathological sup97/149 (65.1%)port or integrated clinicoradiological assessment. These yield denominators should not be conflated with the separately summarized specimen-level isolate records in **Table 2**. Patients without microbiological confirmation showed substantially greater baseline deformity than microbiologically confirmed cases, with a median pretreatment Cobb angle of 33.0 degrees (26.0-37.0) versus 18.0 degrees (8.0-30.3) (P = 0.001). At the specimen level, 95 bacterial isolate records were summarized; Gram-positive cocci predominated, led by *S. aureus* (48 isolate records), followed by Escherichia coli (11), Klebsiella pneumoniae (8), and viridans streptococci (7). No fungal or polymicrobial spinal infections were included in this final pyogenic cohort, and the revised **Figure 2** therefore reports only bacterial taxa.

All patients tested negative for T-SPOT and Brucella agglutination assays. Histopathological examination of all available specimens demonstrated neutrophilic infiltration and purulent cavity formation without caseous necrosis or granulomatous structures, thereby excluding tuberculous or brucellar infection and further supporting the diagnosis of acute pyogenic spondylitis.

To further characterize host inflammatory patterns, an exploratory principal-component analysis integrating baseline WBC, neutrophil percentage, CRP, ESR, PCT, albumin, and IL-6 was performed. The first principal component explained 32.5% of total laboratory variance and was used as an integrated inflammatory-burden score. Scores differed across groups (P = 0.037) but did not increase linearly with structural severity: the median score was lowest in Group C (-0.73), despite the greatest radiographic destruction. This exploratory pattern suggests partial dissociation between structural damage and routine inflammatory intensity in advanced disease.

### 3.3 Dynamic changes in inflammatory markers

**Figure 3:**
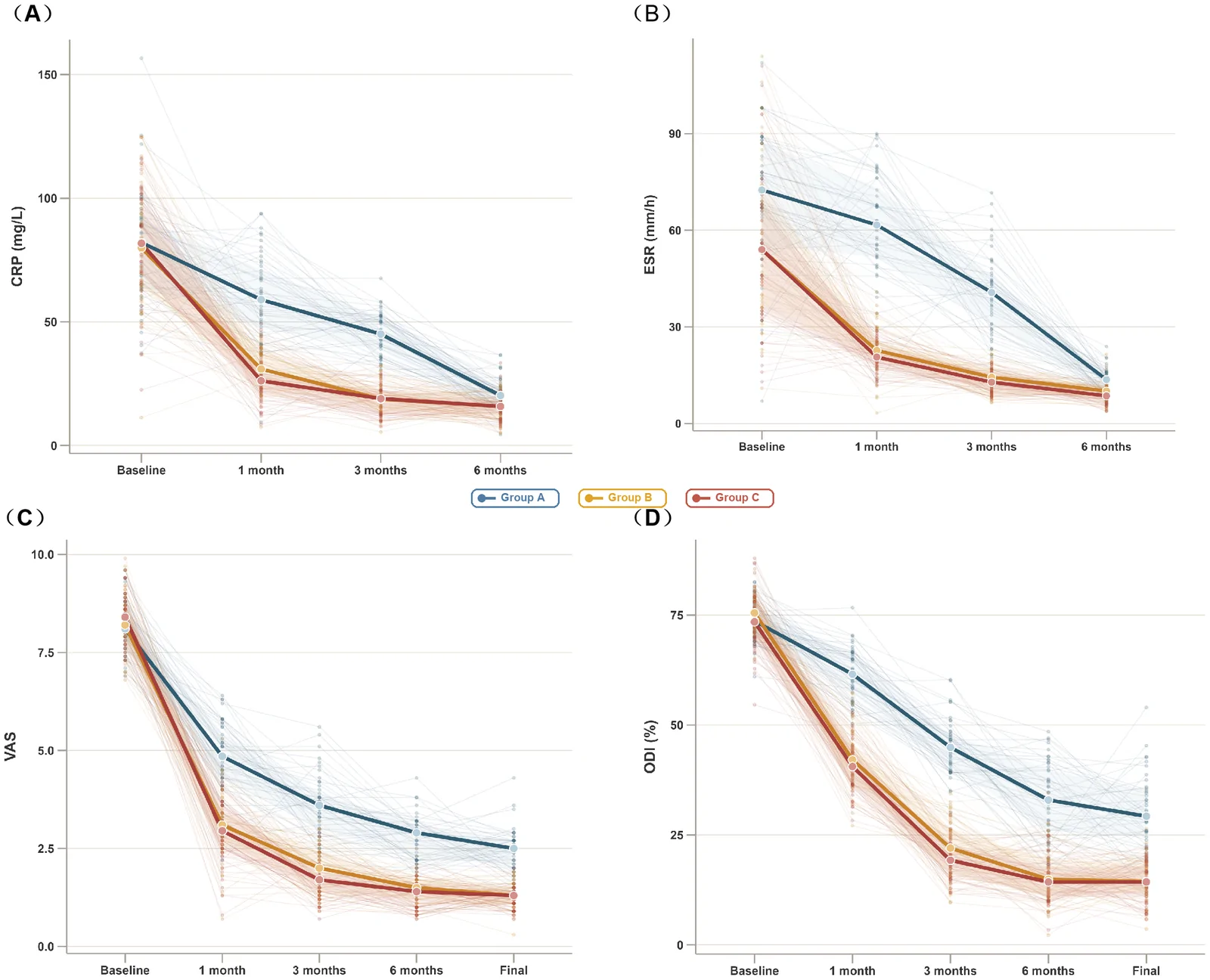
Longitudinal recovery fingerprints of inflammatory, pain, and functional outcomes. **(A)** CRP, **(B)** ESR, **(C)** VAS, and **(D)** ODI trajectories from baseline to follow-up. Blue, gold, and red denote Groups A, B, and C, respectively. Transparent lines indicate individual patients; thick lines and ribbons indicate group medians and interquartile ranges.

C-reactive protein (CRP) and erythrocyte sedimentation rate (ESR) showed a continuous downward trend during treatment in all groups (**Figure 3**, **Tables 3**, **4**); however, the rate of decline differed significantly across disease severity strata.

**Table 3:** Changes in CRP over time in the three groups.

Time point	CRP (mg/L)			*P* value
	Group A	Group B	Group C	
Baseline	55.9(53.1,75.0)	72.6(65.0,81.2)^#*^	88.6(82.1,100.2)^*^	<0.001
1 month	37.8(34.0,44.9)	30.9(23.4,37.0)^#*^	26.2(21.4,29.5) ^*^	<0.001
3 months	26.5(23.0,32.0)	18.8(15,21.9) ^*^	18.9(12.6,21.6) ^*^	<0.001
6 months	14.9(12.0,20.2)	15.8(10.9,19.4)	15.8(12.8,17.9)	0.992

*P < 0.05 compared with Group A.

#*P* < 0.05 compared with Group C.

**Table 4:** Changes in ESR over time in the three groups.

Time point	ESR (mm/h)			*P* value
	Group A	Group B	Group C	
Baseline	59.10 ± 15.38	64.75 ± 18.17**^#^**	73.25 ± 17.84^*^	<0.001
1 month	38.0(35.0,42.3)	22.7(19.6,25.4) ^*^	20.7(17.8,23.9) ^*^	<0.001
3 months	25.8(22.8,31.0)	14.4(9.9,16.6) ^*^	12.9(10.6,15.2) ^*^	<0.001
6 months	13.6(9.1,14.8)	10.1(8.1,11.6) ^*#^	8.6(7.4,10.1) ^*^	<0.001

*P < 0.05 compared with Group A.

#*P* < 0.05 compared with Group C.

At admission, significant intergroup differences were observed in CRP levels. The median CRP values were 55.9 (53.1–75.0) mg/L in Group A, 72.6 (65.0–81.2) mg/L in Group B, and 88.6 (82.1–100.2) mg/L in Group C (*P* < 0.001). A similar pattern was observed for ESR, which increased progressively from Group A (59.10 ± 15.38 mm/h) to Group C (73.25 ± 17.84 mm/h), with Group B showing intermediate values (64.75 ± 18.17 mm/h). These intergroup differences were statistically significant (*P* < 0.001), indicating that inflammatory burden increased in parallel with disease severity.

By 1 month, between-group differences in inflammatory recovery were already evident, with higher early CRP response rates and steeper ESR decline in the surgically treated strata.

By 3 months, inflammatory differences narrowed further, but normalization remained more frequent in Groups B and C than in Group A.

At the 6-month follow-up, intergroup differences in CRP levels were no longer statistically significant, with median values of 14.9 (12.0–20.2) mg/L in Group A, 15.8 (10.9–19.4) mg/L in Group B, and 15.8 (12.8–17.9) mg/L in Group C (*P* = 0.992). However, ESR values remained significantly different among groups, measuring 13.6 (9.1–14.8) mm/h in Group A, 10.1 (8.1–11.6) mm/h in Group B, and 8.6 (7.4–10.1) mm/h in Group C (*P* < 0.001).

Overall, CRP and ESR declined in all three groups. The surgically treated strata showed faster early inflammatory improvement, whereas Group A demonstrated a slower but progressive decline consistent with continued recovery of structurally stable disease. In **Figure 3**, the central label tags identify the blue, gold, and red trajectories as Groups A, B, and C, respectively.

### 3.4 Pain and functional recovery

**Figure 4:**
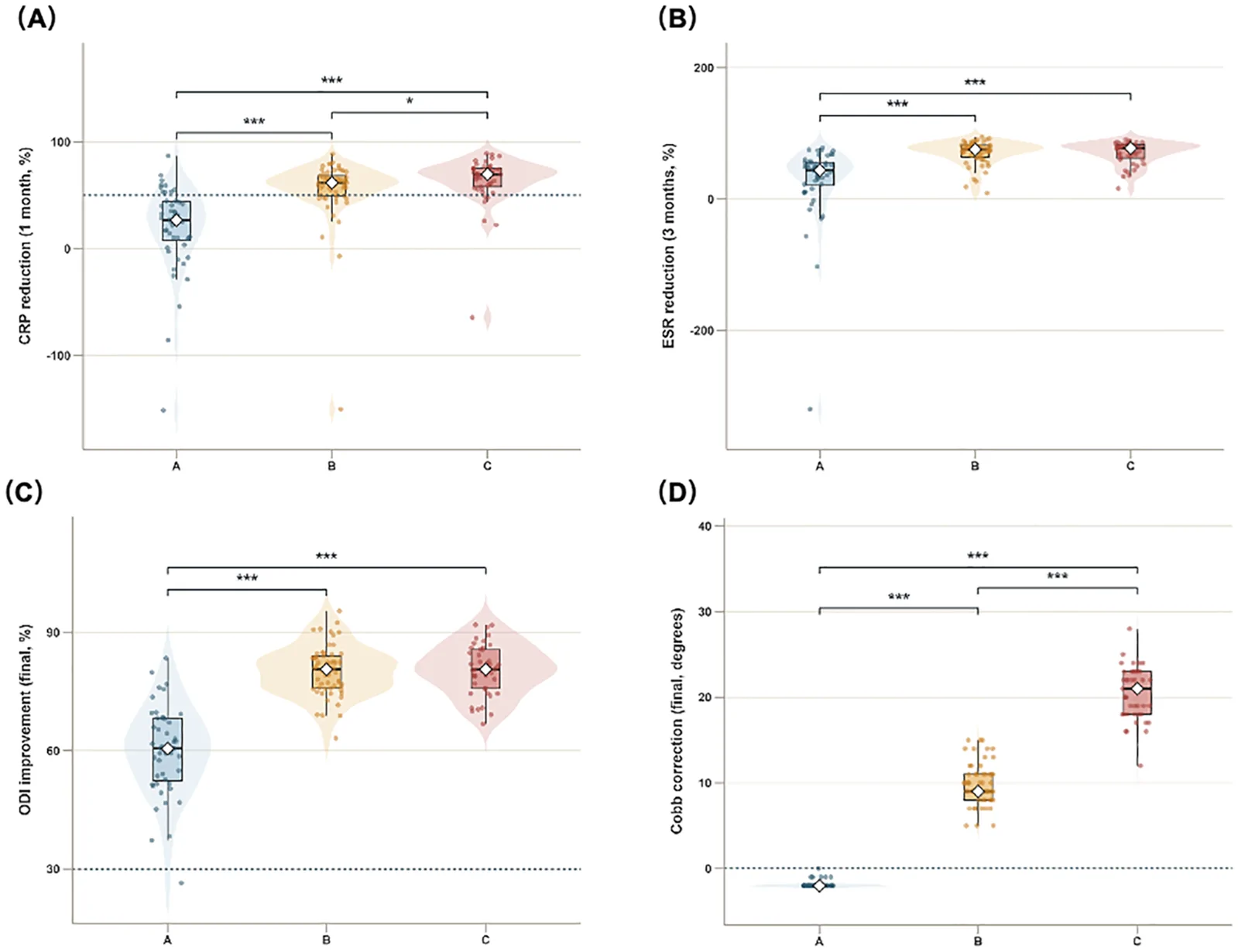
Response distribution atlas across severity strata. Raincloud plots summarize **(A)** early CRP percentage change at 1 month, **(B)** ESR percentage change at 3 months, **(C)** final ODI improvement, and **(D)** final Cobb angle correction. For the laboratory panels, percentage change was calculated as 100 × (baseline - follow-up)/baseline without winsorizing or capping; therefore values below -100% represent interval increases exceeding 100% of baseline. Absolute serial CRP and ESR values are reported separately in **Tables 3** and **4** to provide bounded clinical context. *P < 0.05 and ***P < 0.001.

Pain intensity assessed by the visual analog scale (VAS) and functional disability evaluated by the Oswestry Disability Index (ODI) showed continuous improvement over the course of treatment in all three groups. Baseline VAS values were numerically similar across the severity strata, while ODI values indicated substantial disability in all groups (**Figure 4**, **Tables 5**, **6**).

**Table 5:** VAS scores before and after treatment, median (IQR).

Time point	Group A	Group B	Group C	P value
Baseline	8.1 (7.8-8.5)	8.2 (7.8-8.7)	8.4 (8.1-8.7)	0.207
1 month	4.8 (4.3-5.5)	3.1 (2.7-3.4)	3.0 (2.5-3.6)	<0.001
3 months	3.6 (3.0-3.9)	2.0 (1.4-2.4)	1.7 (1.4-1.9)	<0.001
6 months	2.9 (2.3-3.1)	1.5 (1.2-1.7)	1.4 (1.0-1.6)	<0.001
Final	2.5 (2.1-2.7)	1.3 (1.1-1.5)	1.3 (1.1-1.4)	<0.001

*P* values were calculated using Kruskal-Wallis tests.

**Table 6:** ODI scores before and after treatment, median (IQR).

Time point	Group A	Group B	Group C	P value
Baseline	73.7 (70.7-76.7)	75.5 (72.2-78.1)	73.4 (69.5-78.2)	0.075
1 month	61.5 (57.1-66.1)	42.1 (37.8-45.6)	40.5 (36.5-43.8)	<0.001
3 months	44.9 (40.6-47.8)	22.0 (16.4-26.1)	19.2 (16.4-22.1)	<0.001
6 months	33.0 (26.0-40.0)	14.9 (11.0-19.4)	14.3 (11.1-17.7)	<0.001
Final	29.2 (24.3-34.6)	14.4 (12.0-18.5)	14.3 (10.8-18.0)	<0.001

*P* values were calculated using Kruskal-Wallis tests.

At admission, all three groups showed severe pain and functional impairment. Baseline VAS values were broadly comparable across groups (P = 0.207), while ODI values indicated substantial disability in all strata (P = 0.075). The median VAS score was 8.1 (7.8-8.5) in Group A, 8.2 (7.8-8.7) in Group B, and 8.4 (8.1-8.7) in Group C. The corresponding ODI values were 73.7% (70.7%-76.7%), 75.5% (72.2%-78.1%), and 73.4% (69.5%-78.2%), respectively.

At 1 month after treatment or surgery, pain and functional scores improved in all groups. Median VAS and ODI were 4.8 and 61.5% in Group A, 3.1 and 42.1% in Group B, and 3.0 and 40.5% in Group C (all overall P < 0.001). These data indicate earlier symptom improvement in the surgically treated strata, but the comparison should be interpreted within the severity-stratified pathway rather than as evidence of treatment superiority.

By 3 months, Group A had median VAS and ODI values of 3.6 and 44.9%, whereas Groups B and C had lower values of 2.0/22.0% and 1.7/19.2%, respectively (all overall P < 0.001). The two surgical strata remained broadly similar to each other at this time point.

At 6 months and final follow-up, continued improvement in pain and function was observed in all groups. At the final evaluation, Group A had a median VAS score of 2.5 and ODI of 29.2%, whereas Group B and Group C had median VAS scores of 1.3 and 1.3 and ODI values of 14.4% and 14.3%, respectively. Overall, functional recovery remained internally consistent with the retrospective severity-stratified pathway.

Endpoint-based functional analysis further summarized recovery patterns. A VAS reduction of at least 2 points at 1 month was achieved in 93.8% of Group A and 100.0% of both surgical groups (P = 0.039). At 3 months, ODI reduction of at least 30% was achieved in 85.4% of Group A and 100.0% of Groups B and C (P < 0.001). By the final follow-up, favorable functional status defined as ODI <=20% was observed in 12.5% of Group A, 87.7% of Group B, and 90.9% of Group C (P < 0.001).

### 3.5 Perioperative parameters and radiological outcomes

The two surgical strategies demonstrated differences in perioperative burden and radiological outcomes that were consistent with their corresponding disease severity strata (**Table 7**).

**Table 7:** Operative parameters, radiographic follow-up, and fusion outcomes in surgical groups.

Parameter	Group B	Group C	*P* value
Operative time (h)	2(1.7,2.3)	2.8(2.5,3.3)	<0.001
Blood loss (mL)	416.6(357.7,467)	624.7(521,766.7)	<0.001
Transfusion	11(19.3%)	23(52.3%)	<0.001
Hospital stay (d)	17.77 ± 3.23	19.97 ± 3.91	0.003
ICU admission	2(3.5%)	8(18.2%)	0.019
Time to ambulation (d)	3.28 ± 1.11	5.88 ± 1.26	<0.001
Available radiographic follow-up (weeks)	25.3(20.8,28.5)	26(21.4,29.1)	0.445
Bony fusion rate			
3 months postoperatively	34/57 (59.6%)	21/44 (47.7%)	0.230
6 months postoperatively	50/57 (87.7%)	38/44 (86.4%)	0.840
Final follow-up	57(100%)	44(100%)	1.000

Regarding surgical burden, operative time and intraoperative blood loss were significantly lower in Group B (moderate disease) than in Group C (severe disease). Median operative time was 2.0 (1.7–2.3) h in Group B compared with 2.8 (2.5–3.3) h in Group C, and median blood loss was 416.6 (357.7–467.0) mL versus 624.7 (521.0–766.7) mL, respectively (both *P* < 0.001). The higher transfusion rate observed in Group C (52.3% vs 19.3%, *P* < 0.001) further reflected the increased surgical complexity associated with extensive debridement and reconstruction in severely destructive lesions.

In terms of perioperative recovery, patients in Group B experienced a significantly shorter hospital stay than those in Group C (17.77 ± 3.23 days vs 19.97 ± 3.91 days, *P* = 0.003) and achieved ambulation earlier (3.28 ± 1.11 days vs 5.88 ± 1.26 days, *P* < 0.001). These differences were consistent with the underlying disease severity, as moderate lesions required limited yet sufficient stabilization, whereas severe destructive cases necessitated more extensive debridement and reconstruction, resulting in prolonged perioperative recovery. The intensive care unit (ICU) admission rate was higher in Group C than in Group B (18.2% vs 3.5%, *P* = 0.019), primarily reflecting greater baseline disease severity and surgical complexity rather than an increased incidence of perioperative complications.

With respect to radiological outcomes, the duration of available surgical follow-up was comparable between Groups B and C [25.3 (20.8-28.5) weeks and 26.0 (21.4-29.1) weeks, respectively]. These values represent the cohort-level available radiographic follow-up window rather than a mandatory >12-month inclusion requirement, whereas longer-term one-year images are shown only in representative cases. Cobb angle improved from pretreatment values in both groups and remained stable during the observed follow-up. At the final available radiographic assessment, the median Cobb angle in Group B was 9.0 (8.0-10.0) degrees, whereas Group C improved to 14.0 (13.0-15.0) degrees, despite substantially greater baseline destruction (**Table 8**).

**Table 8:** Changes of Cobb angle before and after treatment.

Time point	Cobb			*P* value
	Group A	Group B	Group C	
Baseline	7.0(6.0,8.0)	19.0(17.0,21.0)^*#^	34.5(32.0,37.0)	<0.001
6 months	8.0(7.0,9.0)	9.0(8.0,10.0)**^#^**	13.0(12.0,14.0)	<0.001
Final	9.0(8.0,10.0)	9.0(8.0,10.0)**^#^**	14.0(13.0,15.0)	<0.001

*P < 0.05 compared with Group A.

#*P* < 0.05 compared with Group C.

**Figure 5:**
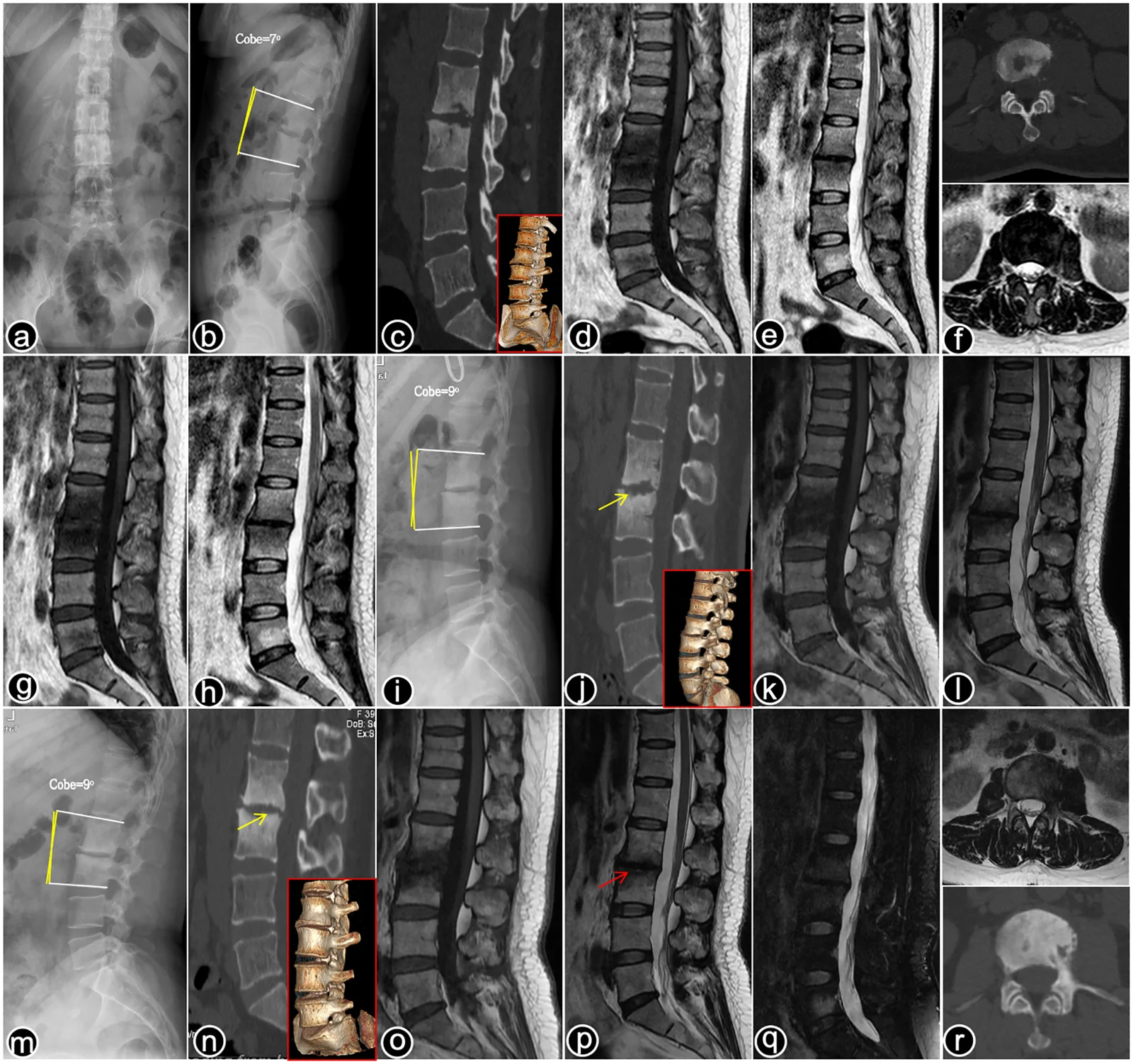
Representative Group A case treated nonoperatively. A 39-year-old woman with L2-L3 pyogenic spondylitis underwent antimicrobial therapy and external immobilization. **(a-f)** Pretreatment radiographs, CT, and MRI show vertebral-endplate destruction, disc-space infection, and paravertebral inflammatory change. **(g-l)** Interval MRI and radiographs demonstrate stable alignment and progressive reduction of inflammatory changes. **(m-r)** One-year imaging in this representative case confirms segmental stability, restoration of structural integrity, and radiographic healing without progression.

**Figure 6:**
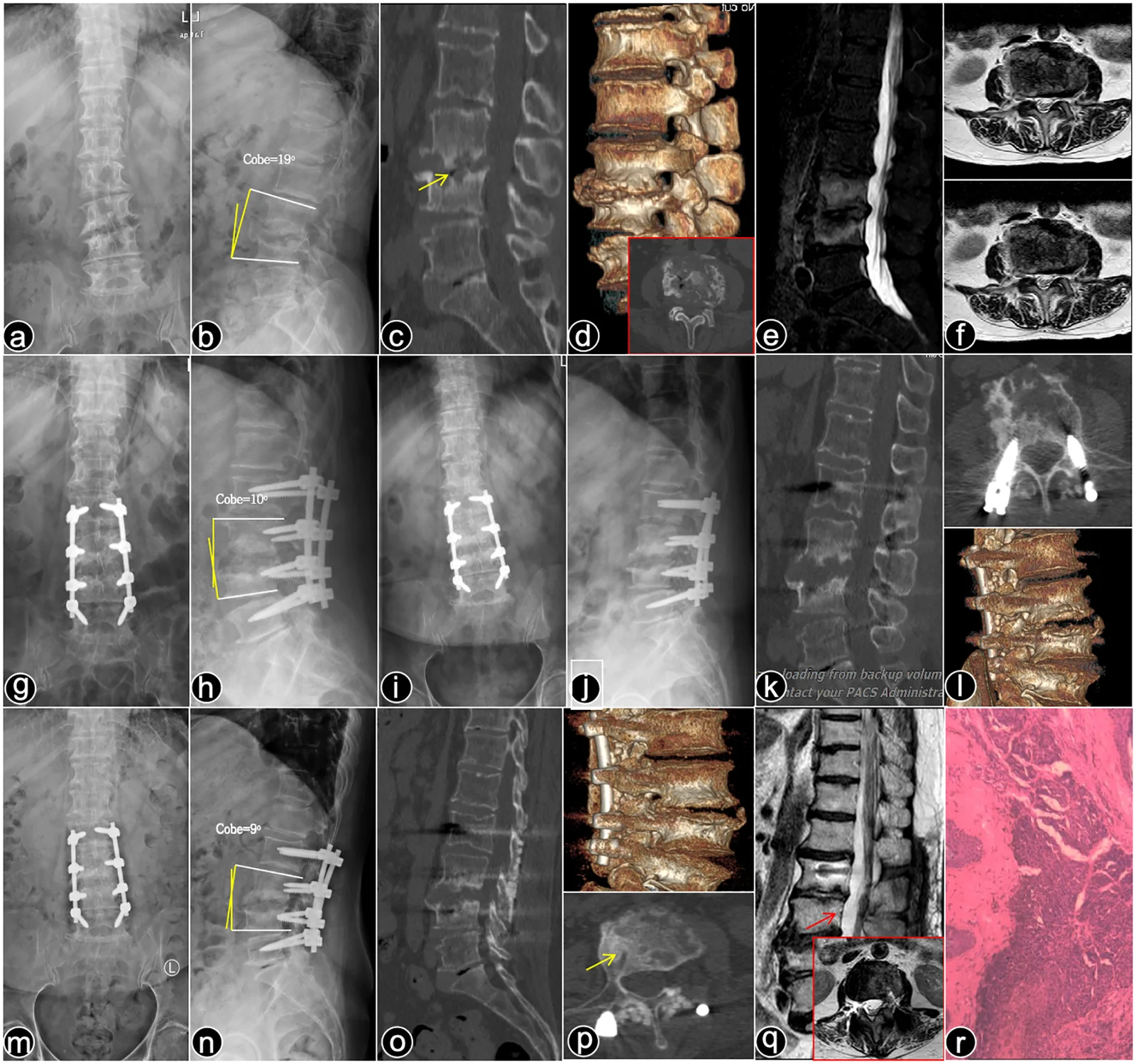
Representative Group B case treated with posterior fixation and fusion. A 61-year-old woman with L3-L4 pyogenic spondylitis underwent posterior pedicle-screw fixation and posterolateral fusion for focal instability. **(a-h)** Pretreatment imaging shows vertebral destruction, local kyphotic deformity, and epidural/paravertebral inflammatory involvement. **(i-l)** Early postoperative and 3-month radiographs/CT show stable implants and new bone formation. **(m-r)** One-year representative imaging confirms mature bony fusion and a patent canal without residual abscess.

**Figure 7:**
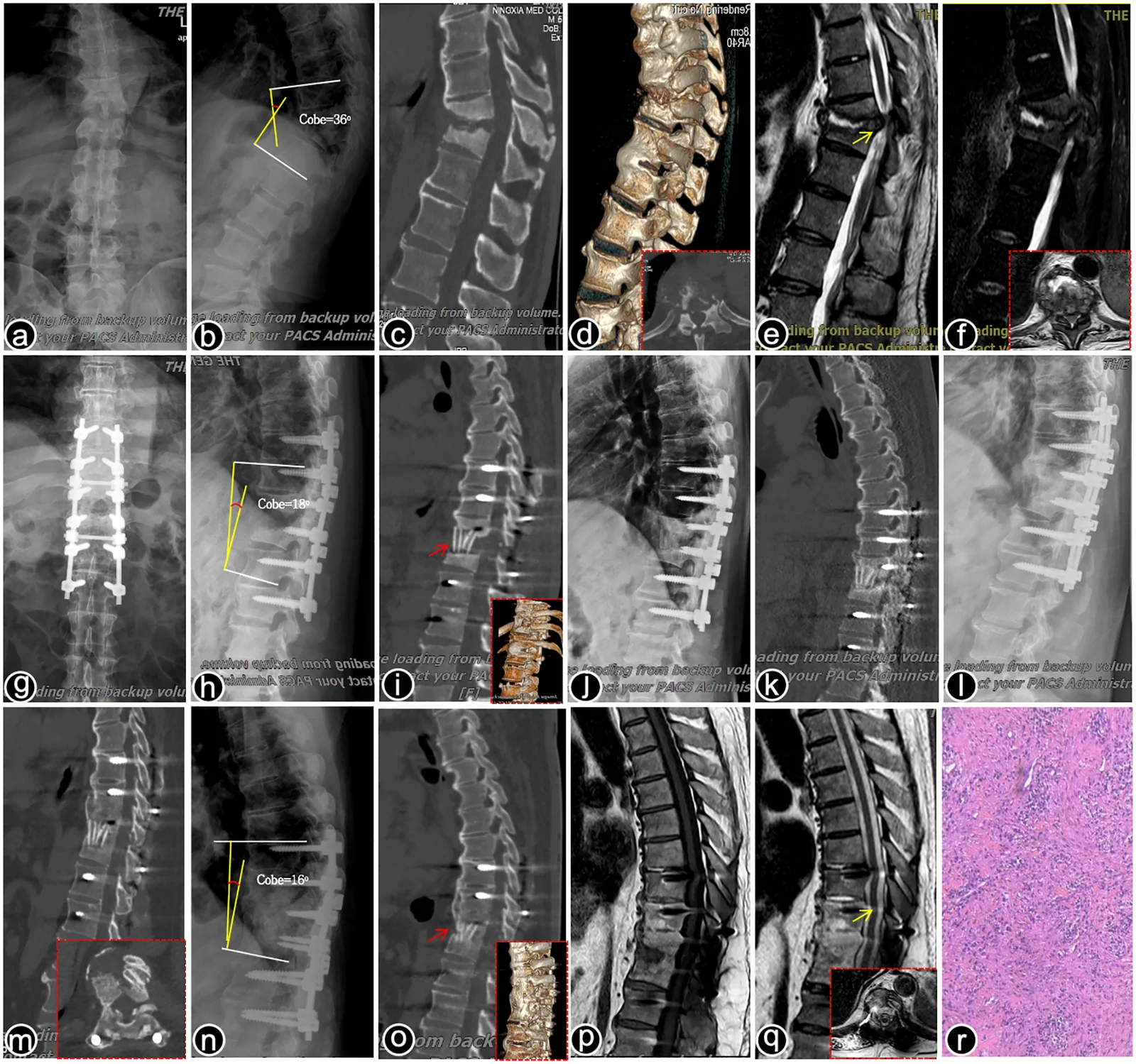
Representative Group C case treated with fixation, debridement, and fusion. A 56-year-old man with T10-T11 pyogenic spondylitis underwent posterior fixation with lesion debridement and interbody reconstruction because of extensive vertebral destruction and neurological risk. **(a-h)** Pretreatment imaging demonstrates marked collapse, kyphotic deformity, epidural compression, and paravertebral infectious burden. **(i-k)** Immediate postoperative and 3-month images show lesion removal, bone graft placement, early bridging bone, and stable fixation. **(l-r)** Representative one-year follow-up confirms durable fusion and maintained correction. These representative one-year images illustrate longer-term radiographic evolution but do not indicate a mandatory cohort-wide >12-month follow-up requirement.

Regarding bony fusion, fusion rates at 3 months postoperatively were 59.6% in Group B and 47.7% in Group C. At 6 months, fusion rates increased to 87.7% and 86.4%, respectively. At the final available follow-up, a fusion rate of 100% was achieved in both groups. No cases of instrumentation loosening or breakage were observed during follow-up in the representative radiological cases (**Figures 5**–**7**).

Overall, perioperative burden, recovery tempo, and radiological improvement followed the expected severity gradient. These findings support the internal coherence of the retrospective severity-stratified pathway while avoiding causal claims about comparative treatment efficacy.

Radiological response endpoints further clarified this pattern. Median final Cobb correction was -2.0° in Group A, reflecting slight progression within a mild range, compared with 9.0° in Group B and 21.0° in Group C. Final Cobb angle stability or correction was achieved in 1/48 patients in Group A (2.1%) and in all patients in Groups B and C (both 100.0%; P < 0.001). Median correction loss from 6 months to the final follow-up was minimal in both surgical groups (0.0° in Group B and 1.0° in Group C), supporting durable maintenance of alignment after fixation and fusion.

### 3.6 Adjusted response analyses

**Figure 8:**
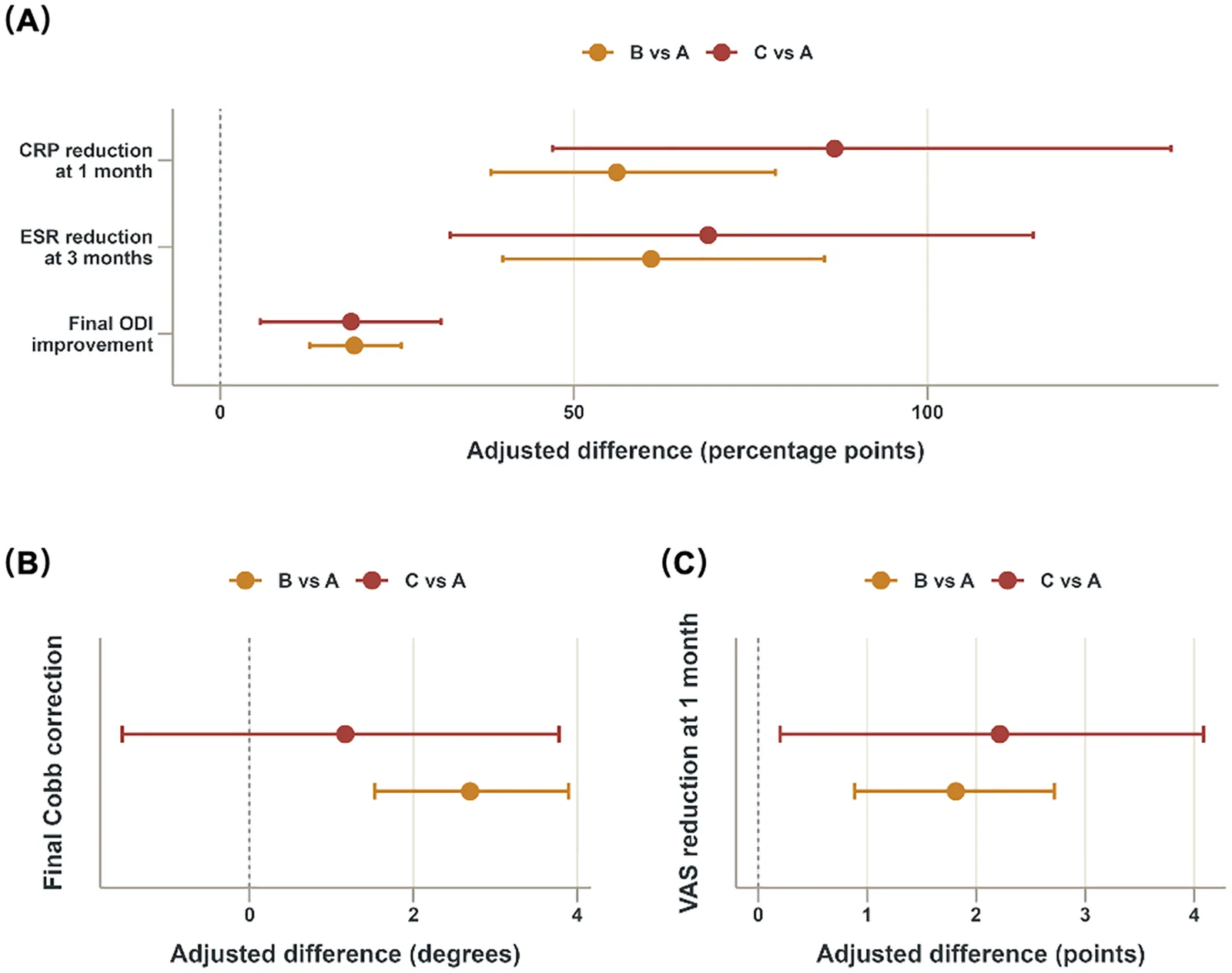
Adjusted treatment-response contrasts versus conservative management. Exploratory standardized linear models compared Group B and Group C with Group A. **(A)** Adjusted differences in CRP percentage change at 1 month, ESR percentage change at 3 months, and final ODI improvement. **(B)** Adjusted difference in final Cobb correction. **(C)** Adjusted difference in VAS reduction at 1 month. For the laboratory panels, these uncapped percentage-change variables were retained only as exploratory adjusted summaries, whereas the serial absolute inflammatory values remain presented in **Tables 3** and **4**. Points indicate adjusted mean differences and whiskers indicate 95% confidence intervals.

Exploratory adjusted models paralleled the unadjusted descriptive patterns but should not be interpreted causally. Compared with Group A, adjusted contrasts for 1-month CRP percentage change, 3-month ESR percentage change, VAS reduction at 1 month, final ODI improvement, and final Cobb correction all showed directionally greater change in the surgically treated groups, particularly when matched to the severity-stratified pathway. These adjusted findings therefore supported the descriptive impression that escalation-matched treatment was associated with coherent recovery within each clinical stratum, although the laboratory percentage-change contrasts remain ratio-sensitive exploratory summaries and are not suitable for causal treatment-effect claims (**Figure 8**).

### 3.7 Representative radiological cases

### 3.8 Complications

Complications occurred across all three groups during treatment or postoperative follow-up. Overall safety was acceptable, and **Supplementary Table 1** now provides the complete all-group complication summary, including recurrence, treatment failure, antibiotic-related adverse events, reoperation, neurological deterioration, implant failure, and mortality.

In Group A, adverse events were mainly related to antimicrobial therapy: three patients developed antibiotic-related liver injury or transient liver-enzyme elevation, and two patients experienced recurrence during follow-up that required extension or escalation of treatment. No progressive structural instability or new neurological deterioration was documented.

In Group B (moderate disease treated with fixation and fusion), complications were infrequent and mainly mild or reversible. One patient developed a superficial surgical site infection that resolved with wound care and antimicrobial therapy. One patient experienced transient postoperative nerve irritation symptoms, and no deep infection or reoperation was required.

In Group C (severe destructive disease treated with debridement and fixation), complication burden was somewhat higher, consistent with greater baseline disease severity and more extensive surgery. Reported complications included one surgical site infection and one cerebrospinal fluid leak; both were managed without reoperation.

During follow-up, no instrumentation loosening, breakage, or implant failure was observed in either surgical group. Recurrent infection occurred in one patient in Group B and one patient in Group C, and no infection-related mortality, new neurological deterioration, or reoperation was recorded in the entire cohort. Overall, the observed safety profile was acceptable within the context of the retrospective severity-stratified pathway.

## 4 Discussion

Pyogenic spondylitis (PS) is a deep, destructive spinal infection that predominantly affects elderly and immunocompromised patients, posing a persistent clinical challenge in balancing effective infection control with timely restoration of spinal stability. Based on nearly eight years of real-world clinical practice at our institution, this study provides a comprehensive analysis of 149 patients with PS. It should be emphasized that the three treatment groups were not established through randomization or direct comparison of competing treatment modalities. Instead, they reflect stratified clinical decision-making based on predefined surgical indications and disease severity in routine practice. Accordingly, differences among the three groups represent the natural spectrum of PS severity rather than comparative superiority among treatment strategies. The primary objective of this study was to evaluate the safety, feasibility, and clinical appropriateness of different interventions within their respective indications, and to summarize a practical tiered treatment framework applicable to real-world settings.

Consistent with recent epidemiological and cohort studies, our results demonstrate a clear trend toward advanced age, male predominance, and substantial medical comorbidity among patients with PS (Son et al., 2023; Thavarajasingam et al., 2023a; Pantel et al., 2024; Conti et al., 2025; Henry et al., 2025; Motoyoshi et al., 2025). The cohort was characterized by a high prevalence of diabetes mellitus, hypertension, hemodialysis exposure, prior spinal procedures, and corticosteroid use, all of which reflect the increasingly complex host profile now associated with spinal infection. Contemporary literature also indicates that healthcare-associated and procedure-related infections constitute a clinically important subgroup, reinforcing the view that PS is no longer merely a sporadic hematogenous disorder but a broader problem of medically complex and surgically exposed patients (Son et al., 2023; Pantel et al., 2024; Bigdon et al., 2025). This evolving host background contributes to delayed recognition, more destructive presentation patterns, and the need for comprehensive evaluation beyond infection eradication alone (Urrutia et al., 2024; Bigdon et al., 2025).

Consistent with this interpretation, the exploratory multivariable inflammatory-burden analysis showed that the integrated systemic inflammatory score did not rise monotonically with disease severity and was lowest in Group C. Although hypothesis-generating, this pattern suggests that advanced PS may sometimes manifest as a structurally severe yet serologically blunted phenotype. Clinically, these data reinforce that deformity, abscess burden, and neurological compromise must remain central in decision-making even when laboratory intensity appears only moderate (Maamari et al., 2022; Crombe et al., 2024; Urrutia et al., 2024; Bigdon et al., 2025; Conti et al., 2025; Haykir Solay et al., 2025; Sato et al., 2025).

Our supplementary analyses add two clinically relevant observations. First, among patients with interpretable microbiological testing records, confirmation was markedly less frequent in Group C than in Groups A and B, and microbiologically negative cases showed substantially greater baseline kyphotic deformity. Second, specimen-level aggregation confirmed a pathogen spectrum dominated by Gram-positive cocci, especially *Staphylococcus aureus*, while Gram-negative bacilli remained a minority. Together, these findings suggest that the most structurally destructive presentations are not necessarily the easiest to microbiologically confirm. Severe deformity in Group C likely reflects later-stage disease after longer symptom evolution, a pattern associated with delayed diagnosis in pyogenic spondylitis (Sato et al., 2025). In such cases, partial antimicrobial exposure before referral and sampling from chronically collapsed, necrotic, or sclerotic vertebral tissue may reduce culture or biopsy yield (Maamari et al., 2022; Haykir Solay et al., 2025). In addition, broader pathogen heterogeneity, including occasional atypical enteric organisms (Hijazi et al., 2024; Zhang et al., 2024), may further limit the performance of routine culture alone. This provides a plausible explanation for why the group with the greatest structural destruction showed the lowest microbiological confirmation rate in our cohort.

Baseline characteristics in this study revealed a continuous gradient from stable disease to moderate instability and finally to severe destructive pathology, which is broadly consistent with recent score-based and framework-based attempts to standardize spinal infection decision-making, including the SITE score and related validation work. The thresholds used in our pathway should therefore be viewed as pragmatic institutional criteria rather than a prospectively validated score. After at least three weeks of systemic antimicrobial therapy, patients in Group A generally exhibited preserved spinal integrity, minimal vertebral collapse, mild Cobb angle changes [7.0 (6.0-8.0) degrees], absence of neural compression, and moderate inflammatory activity, representing a relatively stable disease phenotype. In contrast, Group B patients commonly demonstrated focal endplate destruction, mild instability, moderate vertebral collapse, and intermediate Cobb angle loss [19.0 (17.0-21.0) degrees], often accompanied by mild to moderate neurological symptoms. Group C patients showed severe vertebral collapse, marked Cobb angle loss [34.5 (32.0-37.0) degrees], extensive paravertebral or epidural abscesses, and significant neurological impairment, representing advanced destructive disease. These intergroup differences reflect natural disease stratification rather than arbitrary treatment allocation.

Within this stratified framework, our findings support the clinical plausibility of treatment escalation according to disease severity. In carefully selected patients with structurally stable PS, conservative management can achieve infection control, although close monitoring remains essential. For patients with focal bone destruction and mild instability, posterior fixation and fusion was associated with rapid symptom relief, inflammatory improvement, and high fusion rates, supporting its use as a stabilization-focused strategy for moderate disease. In patients with severe destruction and neurological compromise, debridement combined with fixation was associated with infection control and durable mechanical reconstruction. These associations should be interpreted within the nonrandomized, severity-matched pathway rather than as proof of comparative superiority.

The endpoint-based and adjusted analyses provide additional internal summaries beyond serial median values alone. Early CRP response, 3-month ESR normalization, final functional recovery, and final Cobb stability all showed directionally coherent patterns across the three treatment tiers. These results should not be interpreted as evidence that surgery is universally superior, because treatment allocation was severity based; rather, they indicate that when escalation was matched to mechanical instability and neurological risk, biological, functional, and structural recovery remained clinically coherent within each stratum.

Importantly, our results emphasize that treatment strategies should correspond to disease severity rather than be framed as competing options. Faster inflammatory resolution and improved functional recovery in surgically treated patients do not imply blanket superiority of surgery, but rather reflect appropriate escalation for patients with greater baseline instability or neurological risk. Regarding the safety of instrumentation in the infectious setting, contemporary systematic evidence supports the use of internal fixation when combined with adequate infection control and appropriate antimicrobial therapy (Lim and Kim, 2022; Maddy et al., 2024). In this study, two patients in Group A experienced recurrence during follow-up, highlighting residual heterogeneity even within the mild disease spectrum. These patients were successfully managed by escalation to surgical strategies corresponding to Groups B or C. In Groups B and C, one recurrent infection occurred in each group during a mean follow-up of 24 weeks, without persistent infection or implant failure, further supporting the feasibility of immediate stabilization after debridement or during early surgical treatment (Lim and Kim, 2022; Zhang et al., 2022; Neuhoff et al., 2025).

Based on real-world data from our center, this study proposes and internally evaluates a severity-based stratification and tiered intervention model for PS, systematically characterizing mild, moderate, and severe disease phenotypes and demonstrating the feasibility and safety of corresponding treatment strategies in clinical practice. This framework may complement current score-based and consensus-based approaches, but it requires external validation before broader generalization.

Nevertheless, several limitations should be acknowledged. This study is retrospective in design, with inherent selection bias and limited evidence level; it represents experience from a single center; and the follow-up duration remains relatively limited. The cohort included microbiologically confirmed, pathologically supported, and clinically diagnosed cases, so some diagnostic heterogeneity remained despite the clarified diagnostic framework. Microbiological aggregation included both patient-level and specimen-level summaries, and the inflammatory-burden score and adjusted response models were exploratory rather than externally validated. Because treatment allocation was driven by severity and indication, the present data should not be used to infer comparative treatment efficacy across strata. Future multicenter prospective studies incorporating standardized biopsy protocols, molecular diagnostics, advanced imaging, and longer follow-up are warranted to refine stratification criteria and enable more precise individualized treatment decisions.

## 5 Conclusion

Based on a real-world clinical cohort, this study supports the feasibility of a severity-stratified intervention pathway for pyogenic spondylitis. Patients with different degrees of disease severity showed coherent infection-control, functional, and spinal-stability outcomes when managed with appropriately matched treatment strategies. These findings are descriptive and hypothesis-generating rather than comparative, and they may help inform future diagnostic and therapeutic pathway development.

## Data Availability

The de-identified clinical dataset supporting the conclusions of this article will be made available by the corresponding authors on reasonable request, subject to institutional and ethics requirements.
