# Supplementary material for: Clinical characteristics, microbiological landscape, and severity-stratified management of pyogenic spondylitis: a retrospective cohort study
Source: Front Cell Infect Microbiol. 2026 Aug 28;16:1883513. doi: 10.3389/fcimb.2026.1883513 (PMC13561831; doi:10.3389/fcimb.2026.1883513)
Supplement: Supplementary file 1 [file Table1.docx]

**Supplementary Tables for Revision**

These supplementary tables were finalized against the revised severity-stratified manuscript so that the complication counts, antimicrobial-duration framework, and reviewer responses all use the same denominators and wording.

**Supplementary Table S1. Complete complication summary across the three groups**

| **Event** | **Group A (n=48)** | **Group B (n=57)** | **Group C (n=44)** | **Total (n=149)** |
| --- | --- | --- | --- | --- |
| Recurrence / recurrent infection | 2 | 1 | 1 | 4 |
| Treatment failure requiring escalation | 0 | 0 | 0 | 0 |
| Antibiotic-related adverse events (mainly drug-related liver injury / transient liver enzyme elevation) | 3 | 0 | 0 | 3 |
| Transient postoperative nerve irritation | N/A | 1 | 0 | 1 |
| Surgical site / wound infection | N/A | 1 | 1 | 2 |
| Cerebrospinal fluid leak | N/A | 0 | 1 | 1 |
| New neurological deterioration | 0 | 0 | 0 | 0 |
| Reoperation | N/A | 0 | 0 | 0 |
| Implant failure | N/A | 0 | 0 | 0 |
| Infection-related death | 0 | 0 | 0 | 0 |

Definitions: recurrence indicates renewed clinical or imaging evidence of active infection after initial improvement; treatment failure indicates persistence or progression requiring escalation of management. Counts represent affected patients.

**Supplementary Table S2. Group-wise antimicrobial duration framework aligned with the revised treatment protocol**

| **Group** | **Clinical setting** | **Key timing points** | **IV duration** | **Subsequent pathogen-directed phase** | **Total duration** |
| --- | --- | --- | --- | --- | --- |
| Group A | Medical treatment plus brace immobilization. | Blood cultures before antibiotics when feasible; CT-guided biopsy and/or mNGS if routine cultures were nondiagnostic; no index surgery. | 6 weeks | 6 weeks | 12 weeks |
| Group B | Posterior fixation and fusion for focal instability or mechanical pain without the need for extensive debridement. | Blood cultures before antibiotics when feasible; early fixation after admission evaluation; intraoperative culture and pathology obtained at surgery. | 4 weeks | 6 weeks | 10 weeks |
| Group C | Debridement, reconstruction, and fixation for severe destructive disease, major abscess burden, or neurologic compromise. | Blood cultures before antibiotics when feasible; surgery after imaging and neurologic assessment; intraoperative culture, pathology, and mNGS obtained when available. | 6 weeks | 6 weeks | 12 weeks |

All groups initially received empirical vancomycin plus a third-generation cephalosporin or a fluoroquinolone, followed by pathogen-directed adjustment when microbiological results became available. The durations shown here represent the revised institutional framework summarized in the manuscript and used consistently in the reviewer responses.
